# Common questions and misconceptions about protein supplementation: what does the scientific evidence really show?

**DOI:** 10.1080/15502783.2024.2341903

**Published:** 2024-04-16

**Authors:** Jose Antonio, Cassandra Evans, Arny A. Ferrando, Jeffrey R. Stout, Brandi Antonio, Harry Cinteo, Patrick Harty, Shawn M. Arent, Darren G. Candow, Scott C. Forbes, Chad M. Kerksick, Flavia Pereira, Drew Gonzalez, Richard B. Kreider

**Affiliations:** a Nova Southeastern University, Department of Health and Human Performance, Davie, FL, USA; b University of Arkansas for Medical Sciences, Department of Geriatrics, Little Rock, AR, USA; c University of Central Florida, School of Kinesiology and Rehabilitation Science, Orlando, FL, USA; d Lindenwood University, Exercise and Performance Nutrition Laboratory, St. Charles, MO, USA; e University of South Carolina, Department of Exercise Science, Arnold School of Public Health, Columbia, SC, USA; f University of Regina, Faculty of Kinesiology and Health Studies, Regina, Canada; g Brandon University, Department of Physical Education, Faculty of Education, Brandon, MB, Canada; h Keiser University, Exercise and Sport Science, West Palm Beach Flagship Campus, West Palm Beach, FL, USA; i Texas A&M University, Exercise & Sport Nutrition Lab, Human Clinical Research Facility, Department of Health & Kinesiology, College Station, TX, USA

**Keywords:** Erogenic aid, performance, exercise, supplement

## Abstract

Protein supplementation often refers to increasing the intake of this particular macronutrient through dietary supplements in the form of powders, ready-to-drink shakes, and bars. The primary purpose of protein supplementation is to augment dietary protein intake, aiding individuals in meeting their protein requirements, especially when it may be challenging to do so through regular food (i.e. chicken, beef, fish, pork, etc.) sources alone. A large body of evidence shows that protein has an important role in exercising and sedentary individuals. A PubMed search of “protein and exercise performance” reveals thousands of publications. Despite the considerable volume of evidence, it is somewhat surprising that several persistent questions and misconceptions about protein exist. The following are addressed: 1) Is protein harmful to your kidneys? 2) Does consuming “excess” protein increase fat mass? 3) Can dietary protein have a harmful effect on bone health? 4) Can vegans and vegetarians consume enough protein to support training adaptations? 5) Is cheese or peanut butter a good protein source? 6) Does consuming meat (i.e., animal protein) cause unfavorable health outcomes? 7) Do you need protein if you are not physically active? 8) Do you *need* to consume protein ≤ 1 hour following resistance training sessions to create an anabolic environment in skeletal muscle? 9) Do endurance athletes need additional protein? 10) Does one need protein supplements to meet the daily requirements of exercise-trained individuals? 11) Is there a limit to how much protein one can consume in a single meal? To address these questions, we have conducted a thorough scientific assessment of the literature concerning protein supplementation.

## 1. Introduction

The International Society of Sports Nutrition (ISSN) initially published a Position Stand on Protein in 2007 [1]; subsequently, a revision of that paper came out in 2017 [2]. As of 2023, the 2017 Position Stand on Protein has been cited 890 times. Despite the extensive outreach of the 2017 ISSN position stand paper [2] and numerous reviews and meta-analyzes on protein [3–8], questions and misconceptions regarding protein intake persist. Indeed, some of the most persistent misconceptions include but are not necessarily limited to protein being harmful to one’s kidneys and bones. Other questions surround the limits of protein intake [9,10]. Debate surrounds the optimal amount of protein that can be effectively utilized to promote gains in skeletal muscle mass in a single meal, particularly for individuals engaged in structured resistance training programs. It has been previously suggested (and often accepted) that the rates of muscle protein synthesis (MPS) peak in young adults with approximately 20–25 g of high-quality protein [11]. However, a recent study shows that the limit of protein intake per meal is likely much higher [9]. Furthermore, investigators have posited potential harm associated with high protein consumption (i.e. surpassing the Recommended Dietary Allowance), particularly under specific conditions [12–15]. Consequently, in the management of chronic kidney disease, restricting protein intake may play a significant role [14]. However, Levey et al. found that a 2- to 3-year dietary protein restriction intervention did not yield conclusive effects on renal health in individuals with kidney disease [12]. Moreover, Zhu et al. stated that a “low-protein diet was not significantly associated with improvements in renal function in patients with either type 1 or 2 diabetic nephropathy [15].” It’s important to note that concerns related to dietary protein intake are generally linked to conditions not applicable to exercise-trained individuals. In fact, there is a lack of evidence-based research indicating that high-protein diets pose harm to otherwise healthy, exercise-trained individuals. The development of common questions and misconceptions surrounding protein supplementation stems from the widespread interest and adoption of this dietary practice. As protein supplementation gained popularity, individuals naturally began to seek information to guide their usage. Additionally, as with any emerging trend in nutrition and fitness, misconceptions inevitably arise due to misinformation, conflicting advice, and anecdotal experiences. Therefore, addressing these questions and misconceptions becomes essential to provide accurate information and ensure individuals make informed decisions about incorporating protein supplementation into their diets.

## 2. Is protein harmful to your kidneys?

Perhaps one of the most common misconceptions regarding dietary protein is the purported harm caused to kidney function [16]. It is known that dietary protein intake can modulate renal function [17]. The origin of this misconception stems back to reports which indicated that consuming protein in increased amounts would promote the development of renal disease due to increased glomerular pressure and hyperfiltration [18,19]. It should be noted that issues regarding the potential harm of protein are typically associated with a clinical population. Nonetheless, this does not apply to healthy, exercised-trained individuals. In relation to chronic kidney disease (CKD), Kamper et al. stated that “Daily red meat consumption over years may increase CKD risk, whereas white meat and dairy proteins appear to have no such effect, and fruit and vegetable proteins may be renal protective [20].” Thus, in this clinical population, a specific type of protein (i.e. red meat) may be detrimental. Conversely, dairy and white meat protein may be renal protective [20].

Athletes or exercising populations commonly consume increased amounts of dietary protein, and there is no evidence that this population is at increased risk of renal disease [21,22]. For example, Poortsman and Dellalieux [23] reported that protein intakes in the range of 1.4–1.9 g/kg/day did not impair renal function in a group of athletes consuming increased amounts of dietary protein, while similar outcomes could be concluded from the results of longitudinal studies that have examined the impact of protein supplementation on strength and body composition changes [24–26].

Original research studies have been completed that administered daily intakes of dietary protein greater than the current recommended dietary allowance (RDA) while examining changes in health, glycemic control, body composition, and fat loss [27–34]. Antonio and colleagues [27–30] conducted a series of studies to examine the effect of increased protein intake on health and body composition changes in exercise-trained men and women. Data from these investigations suggest that protein intakes ranging from 3.2–4.4 g/kg/day (4–5.5× greater than the current RDA of 0.8 g/kg/day) are well tolerated with no significant changes in clinical safety markers. For example, one year of a high protein diet (~2.5–3.3 g/kg daily) in resistance-trained males had no effect on blood lipids (i.e. total cholesterol, high-density lipoprotein cholesterol, low-density lipoprotein cholesterol, and triglycerides) [29].

Furthermore, there was no effect on markers of kidney function (i.e. blood urea nitrogen [BUN], creatinine, estimated glomerular filtration rate [eGFR], and the BUN/creatinine ratio) [29]. In a series of case reports in male bodybuilders, protein intakes ranging from 2.6 to 5.8 g/kg daily over a period of two years had no effect on clinical markers of kidney (BUN, creatinine, and eGFR) and liver function (i.e. aspartate transaminase and alanine transaminase) [35].

Another perspective to consider is the popularity of prescribing diets with increased amounts of dietary protein as an effective way to stimulate fat loss and improve one’s body composition in populations generally recognized as being at an increased risk for kidney disease (e.g. dyslipidemia, obesity, hypertension). Consistently, these studies provide evidence supporting the utility of higher protein diets to promote fat loss, improve body composition, and improve health markers without demonstrating any evidence of damage to the renal system. For example, original investigations by Josse [36], Kerksick [31,32], Kreider [33,34], Layman [37–39], Longland [40], Noakes [41], Skov [42] and others have consistently highlighted the favorable impact of increased dietary protein on improving the quality of weight loss and improving various biomarkers reflective of improved glycemic control, cholesterol, and risk for cardiovascular disease. Taken further, Parker et al. [43] and Boden et al. [44] examined the impact of a higher protein diet on health and weight loss outcomes in patients diagnosed with type 2 diabetes, a population known to have varying degrees of compromised renal function. Results from the Parker study [43] reported that increased dietary protein caused greater fat loss in women and greater reductions in low-density lipoprotein cholesterol while failing to instigate any negative health outcomes in this population. In contrast, Boden et al. [44] reported positive improvements in lipid parameters, insulin sensitivity, and hemoglobin A1c. Finally, Moller and colleagues [45] analyzed data from 310 pre-diabetic older (~55 yrs.) adults. They concluded that a higher protein intake was not associated with any changes in creatinine clearance, glomerular filtration rate, or serum creatine. There was no evidence of impaired kidney function after one year of following a diet with a higher protein intake.

The final perspective to consider is the Institute of Medicine [46] and the World Health Organization [47] reports on protein intake, stating there is no evidence linking a higher protein diet to renal disease. Furthermore, a panel charged with establishing reference intakes for Australia and New Zealand [48] stated that no published evidence was available to suggest a diet containing up to 2.8 g/kg/day produces adverse outcomes regarding renal health in athletes and that no known association is available to link elevated protein intake with progressive deterioration of renal function [17].

In summary, individuals engaged in exercise training and who are otherwise healthy can consume protein up to 4 or 5 times the RDA without experiencing adverse effects

## 3. Does consuming “excess” protein increase body fat mass?

Several studies have examined the relationship between high-protein intake and gains in fat mass [49–52]. It is important to note the various definitions for a “high-protein” intake. Operational definitions include protein intakes ranging from 1.0–1.8 g/kg/day, which is greater than the RDA of 0.8 g/kg/day but on the low end of recommendations for active individuals [21,22]. Bray et al. [52] reported the effects of overconsumption of low (5% energy intake), normal (15% energy intake), or high (25% energy intake) protein intakes in healthy but otherwise untrained individuals (16 males and 9 females, 18–35 yrs.). Body mass increased in all groups, and the medium and high-protein intake groups gained more mass compared to the low group; however, all groups experienced similar increases in body fat. However, the normal and high-protein groups gained lean body mass, whereas the low-protein group experienced decreased lean mass. The authors concluded that “calories alone contributed to increased body fat. In contrast, protein contributed to the changes in energy expenditure and lean body mass, but not to the increase in body fat [52].”

Recent studies have found higher protein intakes promote favorable changes in body composition [29,39,40,52–59]. Under hypocaloric conditions, higher protein intakes attenuate the loss of lean body mass and increase the loss of fat mass [39,40,53,54,56,58–60]. This is well documented in healthy weight, active individuals, and overweight/obese individuals. Higher protein intakes (>1 g/kg) correlated with a decreased consumption of refined grains and sugary foods [53]. Similar body composition changes are reported during hypercaloric conditions despite an increase in body mass. Antonio et al. conducted several studies that assessed the effects of a high-protein diet on body composition in exercised-trained individuals [30,61–65]. In one of the studies [30], subjects in the high-protein group consumed 4.4 g/kg/protein, resulting in a significant increase in total energy intake. Body composition and body mass did not change in either the high-protein or control group [30]. A follow-up study compared two different dietary protein intakes (i.e. 2.3 vs. 3.4 g/kg/d) in resistance-trained males and females who underwent a traditional bodybuilding training program [64]. Both groups experienced a similar increase in lean body mass; however, the higher-protein group (3.4 g/kg/d) experienced a greater reduction in fat mass. Furthermore, in an 8-week crossover study in resistance-trained males [28], a high-protein group consumed significantly more protein (3.3 ± 0.8 g/kg/day) and calories than the control group (2.6 ± 1.0 g/kg/day), yet there was no change in fat mass. These studies dispute the notion that excess energy from protein alone promotes gains in fat mass; however, diets high in fats and/or carbohydrates and low in protein tend to promote greater increases in fat mass as well as body mass [66–70].

In summary, a high-protein intake does not necessarily increase body fat mass in exercise-trained individuals. In fact, very high-protein diets in exercise-trained males and females will likely have a neutral effect on body composition unless training is altered. Increases in fat mass are more likely the result of excess energy intake from carbohydrates and fats.

## 4. Can dietary protein have a deleterious effect on bone health?

Protein is involved in numerous metabolic and physiological processes critical to maintaining overall health and performance [2]. As such, athletes are often recommended to ingest a higher protein intake much greater than the general population (e.g. 1.6 g/kg/day vs. 0.8 g/kg/day [2,71]. In contrast to these recommendations, there is a common misconception that higher protein intakes adversely affect bone health [72,73]. This misconception is associated with the acid-ash hypothesis [74]. According to this hypothesis, a dietary pattern abundant in protein and grain foods, combined with low potassium intake, generates an acidic dietary load. Consequently, this triggers heightened net acid excretion (NAE), increased calcium levels in urine, and the release of calcium from the skeleton, potentially playing a role in the onset of osteoporosis [74,75]. Subsequently, an individual ingesting a high-protein diet (especially animal-based) would theoretically have an elevated risk of bone loss over a lifetime. However, there are several limitations to this purported hypothesis. First, the assumption that urinary excretion of calcium comes exclusively from bone is questionable; furthermore, there is evidence that a high-protein [76] diet increases calcium absorbed from foods (which may counter the loss) [77]. Secondly, it is important to consider the diet as a whole; as such, the acidity of the diet may be due to a reduction in other foods, such as fruits and vegetables, which are important for bone health [78].

In contrast, there is evidence that a high-protein diet is beneficial for bone health. [76,79] First, it is important to note that bone tissue comprises protein (50% by weight and 33% by mass) [80]; therefore, an adequate amount of protein is critical for bone health. Furthermore, protein stimulates insulin-like growth factor-1, which is important for bone formation [76,80]. In addition, protein is important in stimulating muscle mass and strength adaptations [2]. Having more muscle and strength will place a greater force or stress on bone tissue and may augment bone adaptations over time [81].

Beyond mechanistic data, there is evidence from high-quality studies and meta-analyzes that protein does not have detrimental effects on bone health. [79,82,83] Antonio et al. [83] examined the effects of a large amount of protein (>2.2 g/kg/day) for six to 12 months in exercising females [83,84]. They found no detrimental effects from protein on whole body or lumbar bone mineral density compared to controls (ingesting ~1.5 g/kg/day) [83,84]. A recent meta-analysis examining milk-derived protein derivatives further noted that protein from dairy (or animal) origin does not support the myth that they are detrimental to bone health [82]. A consensus paper endorsed by the European Society for Clinical and Economical Aspects of Osteoporosis, Osteoarthritis, and Musculoskeletal Diseases and by the International Osteoporosis Foundation [79] noted that variations in protein intakes within normal ranges account for 2–4% of bone mineral variation in adults. They also concluded that higher protein intakes above the RDA (0.8 g/kg/day) are associated with higher bone mineral density, a slower rate of bone loss, and reduced hip fractures (provided there was adequate calcium intake) in older adults.

In summary, there is no evidence that a high-protein diet is deleterious to bone health and may be beneficial.

## 5. Can vegans and vegetarians consume enough protein to support training adaptations?

The misconception that vegetarians (VE) and vegans (VG) cannot consume enough protein to induce favorable training adaptations (i.e. muscle growth, increased strength, reductions in body fat) is rooted in the fact that animal protein sources are recognized as higher quality proteins with a greater concentration of essential amino acids (EAA) [85–91]. Seminal work by Boirie et al. [92] demonstrated that MPS is affected differently depending on the speed of digestion and absorption, as well as the EAA content of the protein source (i.e. whey or casein). In this regard, animal proteins contain a greater amount of EAA (up to ≈ 42% more than plant-based sources), are quickly absorbed, and increase plasma EAA concentrations to serve as a potent stimulator of MPSs. Comparatively, plant-based proteins (i.e. soy, tofu, legumes) are absorbed at slower rates due to their incomplete amino acid profile and lower EAA content [93,94] and thereby do not stimulate MPS to the same magnitude as whey protein [95,96]. For example, Tang and colleagues [95] demonstrated that whey protein produced an 18% (*p* < 0.067) and 31% (*p* < 0.05) greater MPS response than soy protein at rest and post-exercise, respectively. In addition, Yang et al. [97] demonstrated that 20 g and 40 g doses of whey protein effectively stimulated MPS at rest and post-exercise, while the 40 g dose of soy protein post-exercise produced an increase in MPS that was marginally lower (i.e. ≈0.08%/h vs. ≈0.06%/h for whey and soy, respectively). While whey protein triggers the most robust MPS response [92,98–105], emerging evidence suggests that plant-based proteins can elevate MPS above rest levels [94,106–109].

Active individuals and athletes need to consume 1.4–2.0 g/kg/d of protein to maintain positive nitrogen balance and consume protein servings containing at least 6.0 g of EAA [110,111] and 2.0 g of leucine [2,112,113] to optimize MPS to promote favorable changes in muscle mass and strength during training [2]. Importantly, VE and VG may need to increase the amount of plant-based protein consumed to ensure they are obtaining adequate amounts of EAA (especially leucine) that are comparable to animal protein products [88,94,97,114]. One may have to consume up to 53% or 75% more plant-based protein than animal protein to obtain 2.0 g of leucine or 6.0 g of EAA, respectively, depending on the amino acid profiles of the protein source. Consequently, it is important for VE and VG to ensure their protein source(s) contain a sufficient amount of EAA and leucine in a highly digestible format. In addition, VE and VG athletes have been reported to consume less energy and protein compared to their omnivore counterparts [115] and are more susceptible to energy deficits, protein malnutrition, and overtraining [116]. Therefore, emphasis should be placed on VE and VG athletes consuming adequate calories and protein, especially during intense training periods, to maintain a positive protein balance and enhance training adaptation [117].

Evidence indicates that plant-based diets, with supplemental plant protein sources, can increase MPS and augment exercise training adaptions [98,118–128]. With the exception of Volek et al. [99], it appears that plant protein sources can favorably impact body composition and exercise training adaptions when 1) the total daily intake of protein equates to ≈ 1.4–2.0 g/kg/d, 2) the plant protein source delivers ≥ 8–10 g/d of EAA, and 3) the plant protein source delivers ≈2.0 g of leucine [98,100,118–122,124–129]. For example, Hevia-Larrain et al. [128] reported that physically active habitual VG consuming 1.6 g/kg/d from whole foods and supplemental soy (containing sufficient EAA and leucine) for 12 weeks experienced similar body composition and resistance training adaptions compared to a habitual, protein-matched omnivore diet. Both the habitual VG and omnivore groups experienced similar increases in whole-body lean mass (4.4% and 6.2%, respectively), muscle fiber cross-sectional area (data not reported), and one repetition maximum (1-RM) leg press (98% and 102%, respectively). These findings suggest that consuming an exclusively plant-based diet may adequately aid training outcomes when optimal protein ingestion is achieved. Similarly, Candow et al. [120] assessed 27 untrained males and females ingesting either soy or whey protein (three equal doses to meet 1.2 g/kg/d total daily protein) or a placebo for six weeks while following a whole-body resistance training program (4 days/week, 6–12 repetitions at 60–90% 1RM on 6–9 different exercises). Both the soy and whey groups experienced similar increases in lean mass (3.1% and 4.7%, respectively) and 1-RM bench press (13.4% and 14%, respectively) and hack squat strength (34% and 38.6%, respectively) compared to placebo (lean mass: 0.5%; 1-RM bench press: 7.1%; 1-RM hack squat: 19.7%). These findings indicate that resistance training adaptations can be conferred independent of the protein source. Moon et al. [124] also reported that 24 healthy, resistance-trained males consuming 24 g of either rice or a whey protein supplement while following a resistance training program (4 days/week, split body, 3–4 sets of 6–10 repetitions) experienced similar increases in body mass (0.6% vs. 1.4%), lean mass (0.9% vs. 0.7%), and 1-RM bench press strength (3.6% vs. 2.2%) and leg press (6.9% vs. 8.2%) for the rice and whey groups, respectively. Notably, the assessed dose of rice and whey protein delivered ≈10 g of EAA and ≈2.0 g of leucine. This further supports the notion that with careful nutritional planning, plant-based protein sources can trigger favorable training outcomes. Furthermore, Lynch and Collogues [127] demonstrated that untrained males and females supplementing immediately post-training with either soy (19 g) and whey (24 g) protein isolates that were leucine-matched (i.e. ≈2.0 g) experienced similar increases in lean mass (2.5% vs. 3.4%) and isokinetic dynamometer torque for knee flexion (25.3% vs. 33.7%) and knee extension (21.5% vs. 32.3%), as well as similar reductions in body fat percentage (−3.6% vs. −5.4%) for the soy and whey groups, respectively. Last, Banaszek et al. [100] reported 15 trained males supplementing with two 24 g doses of either pea or whey protein pre- and post-high-intensity functional training sessions (i.e. 4×/week of CrossFit at 60–100% 1-RM plus metabolic conditioning) for eight weeks experienced similar increases in 1-RM strength for the squat (6.2% vs. 3.7%, respectively) and deadlift (3.9% vs. 5.2%, respectively). Conversely, Volek et al. [99] reported that less favorable training adaptations (i.e. 1-RM strength and lean mass) would occur when careful planning is not taken to ensure the necessary amounts of EAA and leucine content are delivered per the plant-based source. Whey protein does appear to promote a greater mean change from baseline values (that are not always statistically significant) for body mass, lean mass, 1-RM upper- and lower-body strength, and muscle thickness compared to the plant-based protein source. Collectively, these studies indicate that plant-based protein sources can promote similar exercise training and body composition adaptations to that of animal protein sources when adequate amounts of EAA and leucine are consumed in the diet. Readers are directed to several more comprehensive reviews on this topic [2,94,106,130].

In summary, vegans and vegetarians can meet their total daily energy and protein needs despite the superiority of animal proteins to plant proteins. Vegan and vegetarian athletes generally need to consume ~ 20–40% more plant protein than animal-based protein to provide similar amounts of EAA and leucine, especially during periods of resistance training.

## 6. Is cheese or peanut butter a good protein source?

Many energy-dense food products such as cheese or peanut butter are proudly labeled as good sources of protein, a marketing tactic that might encourage uninformed individuals to consume large quantities of such foods to meet daily protein intake targets. However, these foods often have much higher fat and calorie content with lower protein content per serving compared to popular protein-rich foods such as lean meat or low-fat dairy products like Greek yogurt [131]. In the United States, labeling laws dictate that for a food to be labeled a “good source” of protein, it must contain 10–19% of the daily reference value per serving, which equates to a range of 5–9.5 g of protein. However, consuming a single 2 tbsp serving of creamy peanut butter would not yield sufficient protein (roughly 7 g [132]) to align with evidence-based sports nutrition guidelines such as those put forth by the ISSN, which recommend consuming an absolute dose of protein between 20 – 40 g per meal to maximize MPS and resultant athletic recovery [2]. Standardized US Department of Agriculture data suggests that approximately three servings (roughly 100 g) of peanut butter would be required to meet the threshold of absolute protein intake specified in these guidelines. Though 100 g of creamy peanut butter contains 24 g of protein [132], it also has 49.4 g of fat, resulting in 632 kcal per 100 g. Similarly, 100 g of cheddar cheese contains 23.3 g of protein, 34 g of fat, and 409 kcal [133]. In contrast, 100 g of cooked, skinless chicken breast contains 32.1 g protein, 3.24 g fat, and 158 kcal [134], making it and similar foods a more compelling choice for athletes attempting to consume adequate dietary protein without also ingesting additional unnecessary calories from fat which could increase the risk of unintentional weight gain.

A recent overfeeding study conducted by Antonio and colleagues illustrated the propensity for overconsumption of energy-dense foods like peanut butter to cause weight gain [135]. The researchers recruited 17 exercise-trained males and females to complete a 4-week overfeeding protocol. The participants were instructed to continue their usual dietary and exercise regimens but were also required to consume five additional 16 oz jars of peanut butter across the intervention. Analysis of nutritional intake data from the 14 compliant participants revealed that their dietary fat and total caloric intake significantly increased across the intervention by approximately 46 g and 526 kcal, respectively. The group also experienced a significant increase in fat mass with no concomitant increases in lean body mass or total body water, suggesting that the extra peanut butter intake had a deleterious effect on physique parameters. From a practical perspective, individuals should be wary of consuming large amounts of energy-dense protein sources during periods of caloric restriction when protein needs may need to be higher than usual to minimize the loss of muscle mass [136]. The high caloric content of these foods could make it much more difficult to meet energy intake targets, resulting in stagnation or reversal of diet progress.

In addition to concerns surrounding the energy density and dietary fat content of protein foods, attention should also be given to the quality of the protein source itself. Measures of protein quality and digestibility such as the Protein Digestibility Corrected Amino Acid Score (PDCAAS) and Digestible Indispensable Amino Acid Score (DIAAS) rank plant-based protein sources like peanut butter, nuts, and legumes far below animal sources like dairy and meat [137], meaning that less of the dietary protein contained in the plant-based products is digested, absorbed, and makes its way into the bloodstream. Indeed, recent evidence shows that whole-body net protein balance is appreciably lower after a standardized serving of peanut butter or mixed nuts compared to higher-quality protein sources like beef, eggs, or pork [138]. Therefore, energy-dense plant-based protein foods like peanut butter and nuts should not be used as a primary protein source due to their inferior quality and higher energy density compared to animal protein sources or less energy-dense plant-based products.

In summary, energy-dense foods such as peanut butter and cheese are not ideal sources of protein as they often contain high amounts of fat. Such foods should be viewed as fat rather than protein sources and consumed in moderation to ensure appropriate energy needs are met.

## 7. Does consuming meat (i.e. animal protein) cause unfavorable health outcomes?

Meat is a common part of the human diet in many cultures [139]. It is most commonly harvested from animal skeletal muscle and primarily consists of varying quantities of protein (deemed animal protein), saturated fatty acids, and monounsaturated fatty acids, namely oleic acid [140]. According to the Food and Agriculture Organization, poultry, pork, and beef comprise about 85% of meat consumption worldwide [141].

Epidemiological studies often categorize meats into white meat (including poultry), red meat (including beef and pork), and processed meat (including sausage, cold cuts, etc.) [142]. A 2010 systematic review and meta-analysis of prospective cohort and case-control studies including 56,311 participants found red meat consumption was not significantly related to coronary artery disease. However, processed meat consumption was associated with increased risk [143]. These findings are often attributed to the high content of saturated fatty acids and/or cholesterol; however, there is evidence that saturated fatty acids and/or cholesterol intake are not associated with increased serum lipid concentrations or cardiovascular disease risk [144,145].

Another factor to consider is the potential impact of meat intake on cancer risk. During the cooking process, heterocyclic aromatic amines and polycyclic aromatic hydrocarbons are synthesized from amino acids, creatine, and fatty acids present in meat [146]. It is speculated that exposure to these compounds is associated with cancers, especially of the lung, esophagus, stomach, and colon [147,148]. Several meta-analyzes of prospective cohort studies show an increased risk of cancer in those who consume high amounts of red meat and processed meat [149–154]. Further, a dose-response analysis shows that for each additional 100 grams of red meat and processed meat consumed per day, there is an increase (12 to 35%) in cancer risk [154]. However, these conclusions should be interpreted with caution as much of the data comes from observational studies. Han et al. suggested that the possible absolute effects of red meat and processed meat consumption on cancer mortality and incidence are very small, and the certainty of the evidence is low to very low [155]. Further, Hur et al. concluded that it is difficult to conclude that dietary red meat is the main cause of colorectal cancer [156]. Indeed, multiple factors can impact the etiology of colorectal cancer, such as fruit and vegetable intake, alcohol consumption, smoking, overweight, obesity, and stress [156]. In addition, Yun et al. reported that processed meat intake increases the risk of colorectal cancer rather than other digestive tract cancers; however, no causal relationship was observed between red and white meat intake and digestive tract cancers [157]. Wu et al. found that processed meat may increase lung cancer risk with no evidence of red meat affecting other cancers [158].

It is imperative that we are aware of other dietary or lifestyle factors that can modify the relationship between meat intake and cancer [152,159]. It is also important to note that white meat and fish consumption is not linked to the same negative effects. A recent review of 13 prospective cohort studies showed that white meat is not associated with the incidence of diabetes mellitus but is minimally associated with hypertension, as one included study showed a positive association and was negatively associated with metabolic syndrome [160]. Two meta-analyzes have shown that white meat consumption was negatively associated with colorectal, lung, esophageal, and gastric cancers and not associated with other cancers, including pancreatic and renal [151,161].In addition, fish consumption has been negatively linked to the risk of various cancers, including esophageal [162], colorectal [163], and bladder [164].

In a large multinational investigation published in the American Journal of Clinical Nutrition, the authors suggested that moderate consumption of unprocessed meat is fine, whereas processed meat consumption should be limited. The PURE study (i.e. Prospective Urban Rural Epidemiology (PURE Study) was a cohort of 134,297 individuals enrolled from 21 low-, middle-, and high-income countries. Food intake was recorded using country-specific validated food frequency questionnaires [165]. The primary endpoints were total mortality and major cardiovascular disease (CVD). Processed meat intake was associated with higher CVD risk and total mortality. Conversely, they did not find such an association with poultry and unprocessed meat intake.

As with all studies on meat intake per se, it would be virtually impossible to conduct a randomized controlled trial to establish causality vis-a-vis mortality as it relates to CVD, cancer, etc. Thus, we are left with seemingly contradictory information on this topic.

*In summary, processed meats may have many negative effects on health outcome measures. Nonetheless, one must be cognizant of the effects of other dietary and lifestyle factors. Consuming white meat and fish ostensibly does not pose an increased risk for cardiovascular disease or various cancers and may even reduce the risk of gastrointestinal cancers.*

## 8. Do you need protein if you are not physically active?

Generally, it is believed that only athletes or physically active individuals need protein. However, protein plays a critical role in various physiological processes in the human body, such as protein synthesis, cell signaling, satiety, thermogenesis, and glycemic regulation [166]. The human body comprises approximately 50,000 distinct proteins, of which 65% are found in skeletal muscle [167]. Therefore, adequate dietary protein is essential to maintain muscle, bone, and overall health [166].

The Institute of Medicine [168] advised that all healthy adults require a minimum of 0.8 g/kg/day and an estimated average requirement (EAR) of 0.66 g/kg/day to maintain bodily functions and general health. However, evidence suggests that these recommendations may need to be revised for sedentary individuals [166]. The basis for these protein intake recommendations is drawn from 19 studies that examined nitrogen balance, which measures nitrogen loss (via waste products and sweat) against nitrogen intake (via food consumption) [169]. However, the nitrogen balance method is complex and has overestimated nitrogen retention with an underestimated excretion, thus underestimating protein needs [169,170]. In a follow-up analysis using 28 nitrogen balance studies (including the 19 studies used in the Rand et al. 2003 study), Humayun et al. [171] used a two-phase linear regression instead of the linear regression that was used in the analysis and recommendation of Rand et al. [169] for the 0.8 g/kg/day recommendation for today’s 0.8 g/kg/day recommendation. Humayun et al. [171] suggest that a range of 0.91–0.99 g/kg/day is recommended for sedentary adults, which is 12–20% higher than current recommendations.

Humayun et al. [171] also employed a newer measure, the indicator amino acid oxidation (IAAO) method, to reassess protein requirements. The IAAO method estimates daily protein needs by gauging the efficiency of our body using a specific indicator of essential amino acids in our diet. Their reanalysis of nitrogen balance data, coupled with the IAAO method, indicated that the ideal protein intake for healthy adults ranged between 0.92–1.2 g/kg/day. These values are 15% to 50% higher than the existing RDA recommendations of 0.8 g/kg/day [166,170].

Weiler et al. [172] also highlighted the need for more convincing evidence that current protein intake guidelines (0.8 g/kg/day) are adequate or beneficial for all healthy adults. In addition to the IAAO method, which estimates that the optimal range for protein intake is 15–50% higher than the RDA, studies using the IAAO method used high-quality, easily digestible protein sources [172]. However, Weiler et al. [172] highlights that most adults, even in developed countries, still need to consume high-quality proteins. Therefore, because the results of the IAAO method indicated that daily protein intake should be higher than the current recommendation, and this was done with high-quality protein sources, it can be inferred that those who do not eat high-quality protein sources may need even more than the current recommendation range of 0.92–1.2 g/kg/day by Elango et al. [172]. Furthermore, Vieux et al. [173] suggested that 45–60% of the protein contribution must come from high-quality animal protein sources, as vegan sources may lead to a deficiency of other nutrients such as vitamin B12, iron, calcium, zinc, and omega-3 fatty acids.

Long-term studies have shown that failure to meet protein requirements can negatively impact nitrogen balance, muscle mass, immunity, and functional capacity [174]. A systematic review and meta-analysis by Tagawa et al. concluded that “slightly increasing current protein intake for several months by 0.1 g/kg/d in a dose-dependent manner over a range of doses from 0.5 to 3.5 g/kg/d may increase or maintain lean body mass” [175]. Furthermore, evidence suggests that older adults may need a higher protein intake, as inadequate intake can compromise their health [176]. For example, the Healthy Aging and Body Composition Study showed that older adults who consumed more protein could maintain their lean body mass [177]. Similarly, a survey of 142 older adults also revealed a positive correlation between beef intake and the muscle area of the mid-arm [178]. Older adults exhibit anabolic resistance (i.e. blunted response to dietary proteins), meaning they need more protein than younger adults to maximally stimulate MPS [179]. Anabolic resistance further highlights the need for older people to consume more protein than current recommendations. In light of previously published studies [170–173], the recommended protein consumption should be 1.0–1.2 g/kg per day for optimal health, with 45–60% being from animal protein sources, regardless of the level of physical activity.

In summary, everyone (including sedentary individuals) must consume sufficient dietary protein. Protein serves a variety of important roles that are not exclusive to exercising individuals. In addition, the current evidence suggests that protein intake is the primary modifier of body composition (i.e., higher intakes may produce better body composition).

## 9. Do you need to consume protein ≤1 hour following resistance training sessions to create a muscle anabolic environment?

The notion that protein ingestion has to occur shortly following (≤1 hour) bouts of resistance training likely gained momentum when Esmarck et al. [180] showed that low-dose protein ingestion (10 grams from fat-free milk and soybean) immediately following resistance training sessions (3 days/week) for 12 weeks led to significant increases in muscle cross-sectional and muscle fiber area in healthy older males (*n* = 7; 74 yrs.). Further, immediate post-exercise protein ingestion increased myosin heavy chain (MHC) IIa distribution. However, delaying protein intake for 2 hours post-exercise resulted in no muscle accretion and caused a decrease in MHC IIx distribution (*n* = 6; 74 yrs.). Regarding muscle performance, both protein groups increased strength over time, but the response was more consistent and robust when protein was consumed immediately post-exercise. While this study has been cited > 800 times in the literature, results and generalizability are questionable due to the very small sample size assessed (resulting in inadequate statistical power and possible error), the low dosage of protein supplementation prescribed, and that resistance training failed to produce muscle accretion in the group who delayed protein ingestion 2 hours post-exercise.

Several lines of evidence now refute the critical importance of protein ingestion shortly following (≤1 hour) resistance training sessions to create a muscle anabolic environment. From a mechanistic perspective, Rasmussen et al. [181] found no differences in phenylalanine rate of disappearance (an indicator of muscle protein synthesis) when essential amino acids (6 g) were consumed 1 hour and 3 hours following an acute bout of resistance training in young, healthy adults (*n* = 6; 34 yrs.). Burd et al. [182] showed that rates of myofibrillar protein synthesis were still sensitized (responsive) to 15 grams of protein consumed 24–27 hours post-exercise in young, healthy adults (*n* = 15; 21 yrs.). Thus, even waiting an entire day (post-exercise) to consume a small amount of protein still has muscle anabolic effects.

Furthermore, Wall et al. [183] showed that post-exercise pre-sleep protein (60 g of whey) did not blunt the muscle protein synthetic response to 20 grams of whey protein the following morning (~8 hours separated protein ingestions) in young, healthy adults (*n* = 8; 21 yrs.). Collectively, results across studies indicate that the muscle protein synthetic response to dietary protein remains receptive much longer than 1-hour post-exercise in young, healthy adults.

The necessity to consume protein shortly following resistance training sessions becomes even more arbitrary because pre-exercise protein ingestion produces similar effects. For example, Tipton et al. [184] showed that whey protein (20 g) consumed immediately before or 1 hour following an acute bout of resistance training increased amino acid uptake into skeletal muscle similarly in young, healthy adults (*n* = 17; 27 yrs.). Further, Candow et al. [185] showed that protein ingestion (0.3 g/kg) immediately before or immediately following resistance training sessions for 12 weeks produced similar changes in whole-body fat-free mass, regional muscle thickness, strength, and a surrogate measure of whole-body protein catabolism (urinary 3-methylhistidine excretion) in healthy older males (59–76 yrs.).

In summary, evidence-based research shows that protein ingestion following (≤1 hour) resistance training sessions is not an absolute requirement to produce an anabolic environment in skeletal muscle. What appears more important is the total daily amount of dietary protein consumed. Conversely, it appears reasonable to incorporate protein into one’s post-workout nutrition as a practical approach to fulfill the overall daily protein goal.

## 10. Do endurance athletes need additional protein?

While carbohydrate and lipid oxidation account for the large majority of fuel metabolism during endurance exercise, more prolonged bouts (i.e. >2 h) also begin to enhance the oxidation of amino acids, particularly leucine. Additionally, small intestine injury can result from more prolonged or intense endurance training related to hypoxia [186].In both scenarios, negative whole-body protein balance is a common result [187,188]. Though much of the protein research involving resistance training is often focused on MPS and/or skeletal muscle hypertrophy, protein considerations for endurance athletes must consider more than just these outcomes. Performance and recovery effects are often secondary considerations or overlooked altogether [189], even though protein supplementation may support or enhance the physiological training effects of endurance exercise.

Ingestion of branched-chain amino acids alone has been shown to positively affect time-trial performance and peak power [190] and potentially delay central fatigue through modification of serotonin [191]. However, a combination of protein and other nutrients, especially carbohydrates, appears to have the most pronounced influence on endurance training responses and adaptations with dietary intervention. Meta-analytic evidence revealed average performance improvements, particularly for time to exhaustion, of 9% when ingesting protein plus carbohydrates compared to just carbohydrates [192]. Furthermore, these effects were not simply due to increased energy intake; even isocaloric conditions demonstrated differences. Post-training protein intake also appears to have a favorable effect on glycogen replenishment, which may further influence performance outcomes [189]. This appears to be most impactful when an athlete’s post-training carbohydrate ingestion is suboptimal [193], which is not uncommon in a real-world setting, particularly when there are multiple training sessions per day. There is also evidence that this co-ingestion decreases symptoms of muscle damage [193]. Even including protein in rehydration beverages has been demonstrated to positively impact fluid uptake by the intestines [194].

Training for and competing in marathons presents a unique and real physiological challenge. In one 5-week study, experienced and/or elite marathon runners were supplemented with either 33.5 g/d of maltodextrin or whey protein following each training session leading up to their race [195]. Protein supplementation was found to favorably affect aspartate aminotransferase and alanine aminotransferase as well as markers of muscle damage (i.e. creatine kinase and lactate dehydrogenase) following the marathon compared to carbohydrate supplementation. Both markers of muscle damage were still elevated at one week following the race in the carbohydrate group compared to the protein group. There were also decreases in total cholesterol in the protein group, potentially suggesting that those individuals more effectively converted cholesterol to steroid hormones, which could help explain the differences in recovery [195]. These differences were not just limited to biochemical markers of stress and damage, as the recovery of function within the week following the marathon was also greater in the protein-supplemented group.

Power-endurance athletes, such as soccer players, also appear to benefit from increased protein intake. A milk protein concentrate supplement (80% casein and 20% whey) positively impacted high-intensity running performance in the last 15 minutes of a match compared to an isocaloric carbohydrate supplement when ingested over a 1-week period during the season. [196] The protein supplement also enhanced the recovery of knee extensor concentric strength and endogenous antioxidant responses [196].

Given the more common conversations surrounding higher protein intakes and impacts on the musculoskeletal system, the role of this macronutrient in other systems and physiological adaptations to training often receives a minimized focus. For the endurance athlete, these other functions may be vital to both health and performance. For example, the risk of upper respiratory tract infection is increased in those engaging in high volumes and intensities of endurance training [197]. Daily dietary protein intake of 3 g/kg has been shown to mitigate circulating immune cell disruption during heavy training periods, with values similar to those seen with lighter training, though protein intakes of 1.5 g/kg/d did not. [198,199] These positive effects on immune function have also been seen with BCAA supplementation of 12 g/d. [190]. Along these same lines, high protein intake (~64 g over a 3-hour period after intense endurance exercise) has favorably impacted gene expression related to improved substrate utilization and mitochondrial protein upregulation [200].

In summary, endurance athletes may benefit from higher protein intakes due to their positive effects on glycogen replenishment, enhanced training adaptations and performance, immune system support, and improved recovery markers. Some of these effects are even more pronounced when combined with carbohydrates.

## 11. Does one need protein supplements to meet the daily requirements of exercise-trained individuals?

Exercising populations are recommended to consume protein in amounts ranging from 1.4–2.0 g/kg/day [21,22], and when an individual wishes to maximize their physique through strict dietary restriction, the daily intake and proportion of protein in the diet for these people is recommended to be higher (2.3–3.1 g/kg lean mass/day) [201]. Protein ingestion (3.4 g/kg day) for eight weeks has been shown to improve body composition via an increase in lean body mass coupled with a decrease in fat mass [64]. While this high daily protein intake was achieved through the consumption of whey protein powder [64], Pasiakos and colleagues [202] demonstrated that a daily protein intake 2× and 3× greater than the RDA best maximizes fat loss in the face of a 40% energy deficit over a three-week period in a group of civilian male and female participants. In these situations, total energy intake is a critical consideration, and protein powders offer a pragmatic way to meet increased protein needs while consuming minimal additional calories.

A meta-analysis by Cermak et al. [203] summarized data from 22 separate studies (*n* = 680), concluding that protein supplementation led to significantly greater gains in fat-free mass and lower-body strength. In 2015, Pasiakos et al. [204] published a narrative review and concluded that added protein supplementation favorably impacted changes in muscle strength and hypertrophy in trained individuals but had a lesser impact on untrained people. Finally, a 2018 meta-analysis and meta-regression by Morton and colleagues [8] summarized data from 49 studies representing 1863 participants and concluded that protein supplementation significantly increased maximal strength and muscle fiber cross-sectional area.

In summary, protein supplementation is not required; however, it may provide a convenient adjunct to whole foods to achieve total daily protein requirements.

## 12. Is there a limit to how much protein one can consume in a single meal?

The fundamental requirement for protein intake is the provision of amino acid precursors, particularly the EAA’s, required for protein turnover [205]. Protein turnover is required in all tissues to constantly renew vital body proteins. In muscle, the stimulation of protein turnover is a response to mechano-metabolic stimuli and ensures that damaged, less functional tissue is replaced with more efficient components. While body proteins require a wide range of amino acids, it has long been understood that the stimulation of muscle protein turnover is contingent upon the provision of EAAs [206,207]. It has recently been demonstrated that the delivery matrix and pharmacokinetics of EAAs directly affect the stimulation of muscle protein turnover and MPS [208], with simpler administration formats such as free-form EAA and/or free-form protein combinations, resulting in greater EAA delivery and stimulation of muscle protein turnover. It has been established that both resistance [208] and aerobic [209] exercise stimulates protein turnover in skeletal muscle. Further, exercise sensitizes skeletal muscle to the stimulatory effects of exogenous amino acids [210,211]. Thus, the intuitive conclusion is that muscle activity/exercise requires protein intake for remodeling, performance, and function. However, the question as to a recommended amount of protein is dependent upon several physiological and metabolic factors.

It has been posited that ingesting 20–30 g of high-quality protein, such as whey, will provide adequate stimulation of muscle protein turnover in conjunction with exercise. This recommendation is largely based on two seminal studies by Moore [212] and Witard [213], who demonstrated a maximal MPS response to 20 g of whey protein ingestion in conjunction with exercise. A subsequent study utilizing a greater exercise stimulus (whole-body resistance exercise) by MacNaughton [214] demonstrated a significant increase in MPS with 40 g of protein. Taken together, these data indirectly suggest that greater exercise stress (likely involving more muscle groups) requires greater protein ingestion. This is consistent with recent findings in analogous military-related studies suggesting that caloric deficit, with [215,216] or without [56] concomitant exercise, requires increased protein intake. For example, a 30% caloric deficit requires 35 g of protein intake to ensure whole-body proteostasis and preserve muscle protein turnover [215]. Finally, when considering optimal protein intake, it is important to recognize the importance of the EAA composition of dietary protein. For example, plant-based dietary proteins generally have a lower content of EAA than animal-based dietary proteins. Most studies of dietary protein and exercise have used whey protein, and a greater amount of plant-based dietary protein is likely required to achieve the same results. However, recent work by Trommelen et al. found that ingesting 100 grams of milk protein resulted in a greater anabolic response than 25 grams [9]. This anabolic response was found to be quite prolonged (>12 hours) [9]. This disputes the notion that muscle protein synthesis peaks at ~ 40 grams post-ingestion.

During energy deficit, whole-body proteostasis is maintained at the expense of stimulation of muscle protein turnover [215,216]. These data indicate that the physiologic state dictates adequacy and amount of protein intake. Thus, during a homeostatic, non-stressed state, the mantra of It has been suggested that 20–30 g of high-quality protein is adequate. In contrast, increasing metabolic stress requires greater protein intakes to satisfy both whole-body and muscle requirements. Nonetheless, preliminary data suggests that acute intakes as high as 100 grams result in a greater and more prolonged anabolic response than lower intakes.

In summary, the ideal protein intake is contingent upon the physiologic state. The preponderance of the data suggests that ≥20 g can stimulate MPS in young adults. However, with increasing metabolic stress (e.g. weight loss, greater training volume, etc.), benefits are derived with greater intakes. Moreover, it is not known what the upper limit is for protein intake in a single meal although there is evidence that the acute consumption of 100 grams is indeed utilized by the body.

## 13. Conclusions

1. There is no evidence that consuming dietary protein harms the kidneys of otherwise healthy individuals.
2. In exercise-trained men and women, consuming a high-protein diet either has a neutral effect or may promote the loss of fat mass.
3. There is no evidence that dietary protein has a harmful effect on the bones.
4. Vegans and vegetarians can consume enough protein to support training adaptations.
5. Cheese and peanut butter are inadequate sources of protein.
6. Red meat does not likely cause unfavorable health outcomes; however, processed meat may cause potential harm (e.g.. increased cardiovascular disease risk).
7. Individuals who are not physically active still need dietary protein.
8. Protein ingestion following (≤1 hour) resistance training sessions is not an absolute requirement to produce an anabolic environment. What appears more important is the total daily amount of dietary protein consumed.
9. Endurance athletes need additional protein (i.e., at least twice the RDA) to assist in a variety of issues related to the adaptive response to exercise.
10. One does not need protein powder to meet the daily requirements of exercise-trained individuals. However, treating protein powder differently than typical protein foods (e.g., beef, chicken, milk, etc.) does not make scientific sense.
11. For most individuals, consuming 20–30 grams of high-quality protein is sufficient to induce a significant anabolic response; nonetheless, there is data to suggest that 100 grams can elicit a higher and more prolonged anabolic response.
